# Spontaneous Adrenal Hematoma in a Pregnant Woman; a Case Report

**Published:** 2017-02-24

**Authors:** Mahshid Ghasemi, Ali Akbar Beigi, Faranak Behnaz, Farhad Fathi, Elham Memary

**Affiliations:** 1 Department of Anesthesiology, Ayatollah Taleghani Hospital, ShahidBeheshti University of Medical Sciences, Tehran, Iran.; 2 Department of Surgery, Ayatollah Taleghani Hospital, ShahidBeheshti University of Medical Sciences, Tehran, Iran.; 3 Department of Anesthesiology, ShohadayeTajrish Hospital, ShahidBeheshti University of Medical Sciences, Tehran, Iran.; 4 Department of Anesthesiology, Imam Hosein Hospital, ShahidBeheshti University of Medical Sciences, Tehran, Iran.

**Keywords:** Adrenal glands, adrenal gland diseases, hematoma, hemorrhage, rare diseases, case reports

## Abstract

Spontaneous adrenal hematoma is a very rare condition and its prevalence has been reported to be about 1% in previous studies. Although various causes have been proposed to explain its incidence in existing case reports, the etiology and pathology of this condition is still not known. The present study presents a case of spontaneous adrenal hematoma in a pregnant 31 year old womanwithout history of trauma or other probable risk factors of hemorrhage, presenting to the emergency department with chief complaint of pain in the right flank.Diagnostic measures, imaging and laparotomy,confirmed the diagnosis of spontaneous adrenal hematomafor her.

## Introduction

Spontaneous adrenal hematoma is a very rare condition and its prevalence has been reported to be about 1% in previous studies. Although various causes have been proposed to explain it in existing case reports, the etiology and pathology of this condition is still not known (1, 2). This condition is potentially life-threatening and may lead to primary adrenal insufficiency, especially in bilateral cases. Delay in treatment might lead to shock, adrenal crisis and death (3, 4). This condition has only been available to researchers as case reports in existing literature. In this article, we present a rare case of unilateral spontaneous adrenal hematoma in a pregnant woman.

## Case presentation:

The patient was a 31 year old gravid woman in the 13^th^ gestational week, who presented to the emergency department with complaint of right flank pain. The pain had started gradually from 3 days before and its intensity was increasing. The nature of her pain was pressing and vague and did not diffuse to other parts. It was not related to eating and changing position did not affect its intensity. The patient was not willing to eat but did not complain of nausea and vomiting. She did not have a history of miscarriage, underlying illnesses, using drugs or trauma. Vital signs of the patient on arrival were blood pressure=110/70 mmHg, pulse rate=84/min, respiratory rate=18/min, and temperature=37°C. Examination of head, neck, chest and extremities did not have a pathologic finding. Lung auscultation and chest opening were normal. Cardiac auscultation was normal. Abdomen was soft. There was a little tenderness in the right flank but no rebound and guarding. Distal pulse of the extremities was full and symmetric. Surgery and gynecology consultations were sought. She underwent ultrasonography and in the image,a hyper heteroechoicregion in the anatomic location of adrenal gland was detected with 84×85×116 mm dimensions and 437cc volume, which was suggestive of a mass with internal hemorrhage. In addition, a little free fluid was seen around the subcapsularregion of the liver and the right pleural effusion. The status of the fetus was reported to be normal. To rule out pheochromocytoma, the patient was examined and the results were as follows:

Urine vanillylmandelic acid=10.7 (normal range: 0-13.6)

Urine metanephrine=100 (normal range: <350)

Urine normetanephrine=525 (normal range: <600)

**Figure 1:**
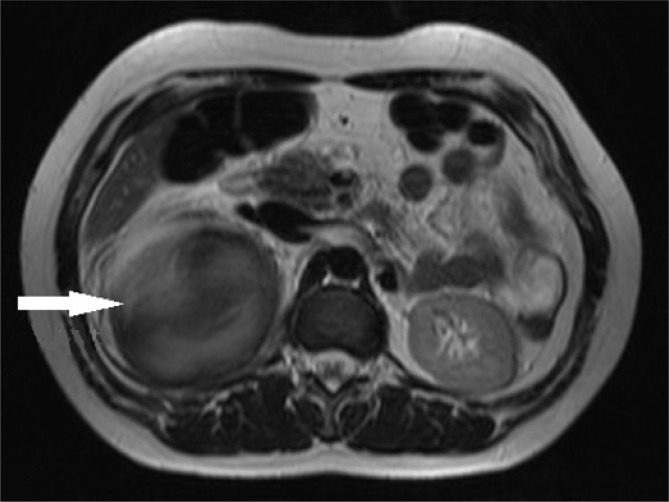
Axial abdominal magnetic resonance imaging cut of patient

During the course of hospitalization, the patient developed a severe abdominal pain and considering the drop in hemoglobin from 12 to 9.7 mg/dL, one unit of packed cells was injected for her. The patient was candidate for abdominal Magnetic resonance imaging (MRI), which confirmed the presence of a mass with internal hemorrhage at the location of right adrenal gland (figure 1). Considering the evaluations done, the patient was finally candidate for laparotomy. A cystic mass with 12 cm diameter was found in the right adrenal and right adrenalectomia was performed and more than 1.5 L hematoma was removed. The sample was sent to pathology unit and the result of evaluating the hematoma was reported. The patient was discharged from hospital after 2 days with good general condition.

## Discussion

Spontaneous adrenal hematoma is a condition etiology and pathophysiology of which is still not identified. Since adrenal gland is a highly vascular organ, it is prone to bleeding (1). Theoretically, it seems that increase in arterial blood flow to adrenal glandand limited venous capacity of this gland, leads to blood stagnation and raises the possibility of thrombosis incidence and results in blood congestion and therefore, hemorrhage in this gland (3). Yet, the considerable point is that in a significant number of reported cases, a trace of coagulation disorders can be seen. Heparin-induced thrombocytopenia, anti-phospholipid antibody syndrome, idiopathic thrombocytopenic purpuramalignancy and prolonged steroid usemay be introduced as risk factors of spontaneous adrenal hematoma (2, 5, 6). In addition, physiological stresses such as burn, sepsis, and post-surgery period should also be considered as potential risk factors (3).

Adrenal hemorrhage does not have specific manifestations and presents in various forms (7, 8). Abdominal pain, vomiting, fever, drop in blood pressure and altered level of consciousness may be seen (3). Although a rare occasion, in bilateral cases, clinical evidence of acute adrenal failure may arise,this may lead to cardiovascular collapse and even death (8).In very rare cases it may also manifest as an abdominal mass, in which case differentiating it from neoplastic diseases would be difficult (6). 

Adrenal hemorrhage, especially in bilateral cases may be life-threatening and therefore, its timely diagnosis is important. Since spontaneous adrenal hemorrhage does not have specific clinical and laboratory manifestation, its clinical diagnosis is very difficult and practically impossible (7, 8). Radiologic imaging is the most effective diagnostic tool and it is interesting that this condition is usually accidentally detected during abdominal computed tomography (CT) scan and is mistaken for adrenal tumor most of the time and its diagnosis is confirmed after surgery (1, 2, 9-11). Color Doppler ultrasonography can reveal the presence or absence of vascularization and help in differentiating spontaneous adrenal hemorrhage from malignancies such as neuroblastoma to some extent. However, it is obvious that abdominal CT scan and MRI are the best diagnostic tools that also help in determining plan of surgery (10, 12). Unfortunately, even these imaging measures lack sufficient specificity for diagnosis of spontaneous adrenal hemorrhage and the diagnosis is only confirmed after adrenalectomy surgery and assessing the pathology of the lesion(11).

Rowe et al., following a study on 18 cases of adrenal hemorrhage, reported that all of the adrenal hematomas demonstrated mass-like configuration with a well-defined, highly variable attenuation, and ovoid morphology on non-contrastCTscan. Some cases had degrees of peripheral enhancement that was either thin and somewhat uniform, or heterogeneous and irregular. None of the lesions showed invasion to the peri-adrenal fat or adjacent organs (11).

In case of life-threatening conditions or evidence of acute abdomen, abdominal surgery has indications. However, in other cases and when there is no confirmed diagnosis, performing surgery may be unnecessary and early steroid replacement therapy may be of priority (3, 10).

In most cases of bilateral spontaneous adrenal hemorrhage, steroids should be prescribed and some even believe that this should be done even in absence of adrenal failure symptoms and especially before surgery. On the other hand, some believe that steroid replacement therapy can be delayeduntil manifestation of adrenal failure symptoms (4). Lack of high evidence studies has made making decisions in these cases difficult and considering the small number of existing references, it might be better to consult an endocrinologist in each case and make decisions specifically for each patient.

It seems that in most cases when facing this condition, especially due to unconfirmed final diagnosis, it is decided to perform surgery. One of the other treatments for this condition is transcatheter arterial embolization (TAE), which is said might prevent unnecessary surgery or at least surgery in emergency conditions that are accompanied by higher mortality rate. Considering the small number of cases with this condition and rare cases of applying this interventional method, its efficiency is still a matter of question. However,in literature,case reports exist thathave shown success of this method in treating spontaneous adrenal hemorrhage (9).
